# Risk Factors for Detection, Survival, and Growth of Antibiotic-Resistant and Pathogenic Escherichia coli in Household Soils in Rural Bangladesh

**DOI:** 10.1128/AEM.01978-18

**Published:** 2018-11-30

**Authors:** Maria Camila Montealegre, Subarna Roy, Franziska Böni, Muhammed Iqbal Hossain, Tala Navab-Daneshmand, Lea Caduff, A. S. G. Faruque, Mohammad Aminul Islam, Timothy R. Julian

**Affiliations:** a Eawag, Swiss Federal Institute of Aquatic Science and Technology, Dübendorf, Switzerland; b Enteric and Food Microbiology Laboratory, International Centre for Diarrhoeal Disease Research, Bangladesh (icddr,b), Dhaka, Bangladesh; c Nutrition and Clinical Services Division (NCSD), International Centre for Diarrhoeal Disease Research, Bangladesh (icddr,b), Dhaka, Bangladesh; d Swiss Tropical and Public Health Institute, Basel, Switzerland; e University of Basel, Basel, Switzerland; Michigan State University

**Keywords:** Bangladesh, CTX-M-group 1, *E. coli*, ESBL-E, enteropathogenic, growth, persistence, households, soils

## Abstract

Soils in household environments in low- and middle-income countries may play an important role in the persistence, proliferation, and transmission of Escherichia coli. Our goal was to investigate the risk factors for detection, survival, and growth of E. coli in soils collected from household plots. E. coli was enumerated in soil and fecal samples from humans, chickens, and cattle from 52 households in rural Bangladesh. Associations between E. coli concentrations in soil, household-level risk factors, and soil physicochemical characteristics were investigated. Susceptibility to 16 antibiotics and the presence of intestinal pathotypes were evaluated for 175 E. coli isolates. The growth and survival of E. coli in microcosms using soil collected from the households were also assessed. E. coli was isolated from 44.2% of the soil samples, with an average of 1.95 log_10_ CFU/g dry soil. Soil moisture and clay content were associated with E. coli concentrations in soil, whereas no household-level risk factor was significantly correlated. Antibiotic resistance and pathogenicity were common among E. coli isolates, with 42.3% resistant to at least one antibiotic, 12.6% multidrug resistant (≥3 classes), and 10% potentially pathogenic. Soil microcosms demonstrate growth and/or survival of E. coli, including an enteropathogenic extended-spectrum beta-lactamase (ESBL)-producing isolate, in some, but not all, of the household soils tested. In rural Bangladesh, defined soil physicochemical characteristics appear more influential for E. coli detection in soils than household-level risk factors. Soils may act as reservoirs in the transmission of antibiotic-resistant and potentially pathogenic E. coli and therefore may impact the effectiveness of water, sanitation, and hygiene interventions.

**IMPORTANCE** Soil may represent a direct source or act as an intermediary for the transmission of antibiotic-resistant and pathogenic Escherichia coli strains, particularly in low-income and rural settings. Thus, determining risk factors associated with detection, growth, and long-term survival of E. coli in soil environments is important for public health. Here, we demonstrate that household soils in rural Bangladesh are reservoirs for antibiotic-resistant and potentially pathogenic E. coli strains and can support E. coli growth and survival, and defined soil physicochemical characteristics are drivers of E. coli survival in this environment. In contrast, we found no evidence that household-level factors, including water, sanitation, and hygiene indicators, were associated with E. coli contamination of household soils.

## Abstract (precis)

Soil may represent a direct source or act as an intermediary for the transmission of antibiotic-resistant and pathogenic Escherichia coli strains, particularly in low-income and rural settings. Thus, determining risk factors associated with detection, growth, and long-term survival of E. coli in soil environments is important for public health. Here, we demonstrate that household soils in rural Bangladesh are reservoirs for antibiotic-resistant and potentially pathogenic E. coli strains and can support E. coli growth and survival, and defined soil physicochemical characteristics are drivers of E. coli survival in this environment. In contrast, we found no evidence that household-level factors, including water, sanitation, and hygiene indicators, were associated with E. coli contamination of household soils.

## INTRODUCTION

The relative importance of different routes of enteric disease transmission is not well understood (1), even for the model organism, traditional indicator of fecal contamination, and frequent pathogen Escherichia coli (2). E. coli transmission is traditionally considered to occur via the fecal-oral route (2) or through interactions with environmental compartments contaminated with feces (i.e., water, hands, and soils) (3, 4). Interactions of infected, colonized, and susceptible hosts (human and animal) with environmental compartments play an important role in enteric disease transmission, and in E. coli specifically (3). E. coli pathotypes infect multiple host species (i.e., humans, ruminants, and chickens) that are often in close contact and share space, especially in low- and middle-income countries (LMICs) (1). Understanding the transmission of E. coli pathotypes is important in developing effective water, sanitation, and hygiene (WASH) interventions (3).

Research and WASH interventions have primarily focused on improving microbial quality in water and food. Recent evidence suggests that other reservoirs (i.e., hands and soil) also act as intermediaries of transmission either directly (i.e., hand-to-mouth contacts and soil ingestion) or indirectly (through interactions with other environmental matrices) (5–9). Effective interventions may need to limit transmission through microbial control of these additional reservoirs. Indeed, of the three recent randomized controlled trials of WASH investments (WASH Benefits Bangladesh, WASH Benefits Kenya, and SHINE Trial in Zimbabwe), only the WASH Benefits Trial in Bangladesh showed reductions in child diarrheal disease (10–13). The failure of WASH investments to improve health may be partially attributed to the failure of the interventions to adequately reduce enteric pathogens and fecal contamination in environmental compartments, including soils (14).

Pathogen transmission via soil is particularly relevant for children given the high rates of observed soil ingestion in LMICs (5, 6, 15). For example, one study in rural Zimbabwe estimated that a 1-year-old child may ingest more than 20 g of soil per day as a result of both active soil ingestion and mouthing episodes with soil-contaminated hands (8). Furthermore, the soil in households in LMICs is frequently found to contain high concentrations of E. coli (6, 16, 17). The detected E. coli strains include multiple intestinal pathotypes, as evidenced by a study in Tanzania (17), indicating that soils may be contributing to pathogenic E. coli transmission in these settings. Similarly, soil may play a role in the transmission of antibiotic-resistant E. coli strains, which have also been detected in soils (18). The consumption of fresh produce grown in soils contaminated with E. coli also represents a health risk (19), as it has been shown that even after washing, the concentration of bacteria can remain high (20).

The high concentrations of E. coli in soil may be linked to soil-associated growth and/or survival. Growth dynamics of E. coli have been studied in soils, sand, and sediments to demonstrate potential limitations of E. coli as an indicator of fecal contamination (21–23). For example, Ishii et al. hypothesized that E. coli strains are naturalized to the soil environment as stable members of the soil microflora based on isolation of the same E. coli genotypes at the same location repeatedly over 1 year (21). In addition, the phylogenomic analysis of five Escherichia clades (isolated primarily from environmental compartments), which are phenotypically indistinguishable but genetically distinct from E. coli (24, 25), has strengthened the view that there are environmentally adapted lineages. This was previously suggested by Byappanahalli et al. after observing DNA fingerprints of E. coli strains from soils distinct from those of strains from animal sources (26). The existence of environmentally adapted lineages suggests the possibility of strain-specific adaptation for survival and/or growth in soil. However, it is also clear that survival and/or growth are influenced by environmental factors, including temperature, water content, nutrient availability, soil texture, pH, solar radiation, and the presence of soil indigenous microflora (27–31).

In recent years, more attention has been given to the role that environmental matrices play in pathogenic E. coli transmission. However, fundamental questions remain about the importance of E. coli adaptability, survival, and growth in the environment. In this study, we evaluate E. coli ecology in soils collected from Mirzapur, Bangladesh, with the focus on soil as a reservoir for E. coli transmission. Specifically, we investigated risk factors associated with the detection and concentration of E. coli in household soils in rural Bangladesh. We also assessed the survival and growth dynamics of antibiotic-resistant and potentially pathogenic E. coli in soil microcosms to further highlight mechanisms by which soil intrinsic properties influence E. coli detection, survival, and/or growth.

## RESULTS AND DISCUSSION

### Household characteristics, animal ownership, and feces management.

Survey data on household characteristics, including animal ownership and feces management, allowed a comparison of the study site to previous studies and provided insight into the importance of household-level factors that may contribute to increased E. coli in the soil. We found that the enrolled households were generally more affluent, with respect to durable assets and animal ownership, than typical rural households in Bangladesh, as described by a 2014 demographic health survey (32) (Table 1; see also Table S1 in the supplemental material). For example, the enrolled households reported higher ownership of electricity, televisions, mobile phones, refrigerators, wardrobes, fans, cows/bulls, and chickens/ducks (Table S1). To assign households to wealth quartiles based on durable assets, animal ownership, and household characteristics, composite wealth indices were constructed using principal-component analysis. Indices ranged from −0.39 to 4.52 and correlated moderately with self-reported monthly expenditures (Spearman’s ρ = 0.53, *P*< 0.001). Wealth quartiles were defined using k-means clustering, with 33%, 21%, 29%, and 17% of the households categorized in the poorest, second, third, and wealthiest quartiles, respectively (Table 1). Wealth quartiles represent variation in wealth only among households enrolled in the study.

**TABLE 1:** Characteristics of the 52 households in Mirzapur, Bangladesh, enrolled in this study and *E. coli* counts in soil

Characteristics	No.	%	E. coli log_10_ CFU/g (dry soil)		Significance (*P* value)	Test
			Mean	SD		
Wealth quartile					0.96	Kruskal-Wallis test by ranks
First (poorest)	17	33	1.27	0.80		
Second	11	21	1.25	1.05		
Third	15	29	1.11	0.69		
Fourth (wealthiest)	9	17	1.41	1.08		
Monthly expenditures^*a*^					0.86	Spearman's rank correlation (ρ = −0.03)
Toilet/latrine					0.29	Kruskal-Wallis test by ranks
Improved, basic	37	71.2	1.14	0.79		
Improved, limited	13	25	1.46	1.00		
Unimproved	2	3.8	1.81	1.17		
Toilet was serviced/pit emptied:					0.61	Kruskal-Wallis test by ranks
In the last month	3	5.8	1.17	0.83		
Between 1 month and 1 yr	18	34.7	1.10	0.91		
Between 1 yr and 5 yrs	4	7.7	1.54	1.00		
Never	27	51.9	1.31	0.84		
Toilet age					0.15	Spearman's rank correlation (ρ = −0.21)
Visible feces observed around the toilet/latrine					0.13	Wilcoxon signed-rank test
No	22	42.3	1.59	1.08		
Yes	30	57.7	1.00	0.55		
Soap present in toilet/latrine					0.76	Wilcoxon signed-rank test
No	46	88.5	1.25	0.86		
Yes	6	11.5	1.26	0.91		
No. of users					0.60	Kruskal-Wallis test by ranks
1–5	27	51.9	1.12	0.74		
6–10	22	42.3	1.41	1.00		
>11	3	5.8	1.15	0.81		
No. of users <5 yrs old					0.43	Wilcoxon signed-rank test
0	34	65.4	1.21	0.81		
≥1	18	34.6	1.31	0.96		
Incidence of diarrhea					0.36	Kruskal-Wallis test by ranks
In the last 7 days	6	11.5	1.92	1.0		
Within last month	8	15.4	1.00	0.53		
Within last 6 months	7	13.5	1.12	0.59		
In more than 6 months	31	59.6	1.21	0.92		
Incidence of respiratory symptoms in last 7 days					0.83	Wilcoxon signed-rank test
No	17	32.7	1.24	0.84		
Yes	35	67.3	1.25	0.878		
No. of chickens/ducks					0.64	Wilcoxon signed-rank test
<10	37	71.2	1.29	0.90		
≥10	15	28.8	1.15	0.77		
Cattle					0.31	Wilcoxon signed-rank test
No	26	50	1.40	0.96		
Yes	26	50	1.10	0.74		

a Self-reported in response to the questionnaire.

Among the enrolled households, sanitation was generally improved relative to the status reported in a 2014 demographic health survey for rural Bangladesh (32). For example, 71.2% of the households in this study had improved latrines with basic sanitation services, compared to 43.6% in rural Bangladesh (Tables 1 and S1). Nevertheless, visible feces were observed in 57.7% of the toilets/latrines, while only 11.5% had soap. Toilet/latrines were shared among 1 to 5 people in 51.9% of the households, while 48.1% were shared among 6 to 19 people (Table 1). Among the 18 households with children under 5 years (Table 1), 55.6% reported that the child uses the toilet, and none reported the use of diapers. The most common way (44.4%) to manage the child feces was disposal into the garbage. All the households had domestic animals (Tables 1 and S1), and all reported that the animals defecate on the ground inside the household plot. Diarrhea (defined as 3 or more episodes of loose/watery stool per day) or respiratory symptoms (runny nose and cough) 7 days prior to the interview date were reported in at least one member of the household in 11.5% and 67.3% of the instances, respectively (Table 1).

### E. coli concentrations in household soils.

Presumptive E. coli was isolated from 44.2% (*n* = 23/52) of the soil samples collected in the household plots, with an average ± standard deviation of 1.95 ± 0.88 log_10_ E. coli CFU/g dry soil and a maximum count of 3.86 log_10_ E. coli CFU/g dry soil. The mean and maximum E. coli concentrations observed in this study were similar to those in other studies in Tanzania and Zimbabwe (17, 33) but lower than those from a previous study in rural Bangladesh (16). Species identification using the API-20E system confirmed E. coli in all 23 soil samples (100%). The majority of the isolates (21/23) were identified with a confidence level of >95%, while 2/23 showed lower discrimination confidence. The API-20E results also indicated high phenotypic diversity among the isolates, as indicated by 10 unique biochemical profiles. Random amplified polymorphic DNA (RAPD) confirmed a high degree of genetic diversity among the soil isolates. All isolates showed unique fingerprint patterns, and only nine isolates clustered together in three RAPD types with similarity greater than 80% (RAPD types G, I, and K; Fig. S1). PCR detection of the E. coli gene *uidA* from DNA extracted directly from the soil samples increased, albeit not substantially, E. coli detection from 44.2% to 57.7% (*n* = 30/52). This result indicates that the culture method used for isolation was able to recover E. coli in the majority of soil samples where E. coli DNA was detected.

### Associations between soil characteristics and E. coli concentrations in soil.

We evaluated E. coli concentration associations with different soil characteristics measured, as these varied across households. Soil water content was significantly correlated with the concentration of E. coli in soils (Spearman’s ρ = 0.48, *P* = 0.0003; Table S2), consistent with previous studies (16, 28, 33). Water content in the 52 soil samples varied between 9.8% and 38.4%, with a mean ± standard deviation of 20.8% ± 7% (Table S2). The only other soil physicochemical parameter that was found to be associated with E. coli concentrations was the percentage of clay, with an inverse correlation (Spearman’s ρ = −0.47, *P* = 0.0095; Table S2). The mechanism explaining the inverse relationship between E. coli concentration and clay is unclear. In agreement with our findings, Lang and Smith reported higher background concentration of E. coli in a sandy loam soil (73% sand, 19% silt, and 8% clay) than in a silty clay soil (11% sand, 53% silt, and 36% clay) (34). In contrast, previous studies have observed a higher proportion of bacteria (35) and preferential attachment (36) in the clay fraction of soil than in the other fractions. E. coli O157 was also observed to survive longer in loam and clay soils compared to a sandy soil (37). In addition, Brennan et al. showed that the addition of different clay minerals (clay mineral composition varies among soils) influenced other physicochemical soil properties and differentially affected the survival of enteropathogens (38). The contrasting results may also be due to differences in the methods for bacterial recovery, suggesting that further evaluation of the methodology for E. coli enumeration in soils may be warranted. Correlations with other soil properties (field capacity, permanganate oxidizable active organic carbon, active organic carbon, total nitrogen, and percentage of silt and sand in soil) were not significant (Table S2).

### Associations between household characteristics and E. coli concentrations in soil.

Differences in household characteristics, WASH indicators, diarrhea/respiratory symptoms, and animal ownership could not account for differences in E. coli concentrations in soils (Table 1). This study was designed to include the same number of households with ruminants and without ruminants in order to determine if ruminants significantly increase E. coli contamination in the household soil environment. Our results indicate that the presence of ruminants in the vicinity of the household plot was not associated with E. coli concentration (Wilcoxon signed-rank test; *P* = 0.31) (Table 1) or the presence/absence of culturable E. coli in soils (Fisher’s exact test; *P* = 0.58). In previous studies, the presence of roaming animals and animals in general has been associated with higher levels of E. coli in soils, although the differences in concentrations were low (0.22 and 0.54 log_10_ CFU/g dry soil, respectively) (16, 33). Our study was likely underpowered to observe the significance between WASH indicators (except the presence and absence of ruminants) and the concentration of E. coli in soils at the previously observed effect size. For example, all the households included in our study had chickens and other domestic animals that defecate inside the household plot and potentially contribute to contamination of soils by E. coli. Indeed, not only a ruminant-associated molecular source tracking (MST) marker (BacR) but also an avian-associated MST marker (avian-GFD) have been detected in soil samples in rural Bangladeshi households (39). Nevertheless, the absence of a clear relationship between E. coli contamination in soils and household-level factors stands in contrast to the relationships observed with soil properties (moisture content and clay percentage).

### Antibiotic resistance pattern and presence of extended-spectrum beta-lactamase genes.

The level of susceptibility to a panel of 16 antibiotics was evaluated among the 175 E. coli isolated from soil samples (*n* = 23) and fecal samples from humans (*n* = 50), chicken (*n* = 51), and cattle (*n* = 51). Overall, 42.3% of the isolates were resistant to at least one antibiotic category, and 12.6% were resistant to 3 or more antibiotic categories, thus classified as multidrug resistant (MDR) (Table 2). Resistance to tetracycline (27.4%) and ampicillin (20.6%) was predominant, followed by resistance to nalidixic acid (12.6%) and trimethoprim-sulfamethoxazole (10.3%). Resistance to other antibiotics was less prevalent (1.1 to 5.7%), while no resistance to piperacillin-tazobactam, meropenem, imipenem, or amikacin was observed (Table S3).

**TABLE 2:** Distribution of the 175 antibiotic-susceptible and -resistant *E. coli* isolates by source

Source	No. (%) of susceptible isolates	No. (%) of resistant isolates to one antibiotic in 1 to ≥3 antibiotic categories^*a*^		
		1	2	≥3^*b*^
Soil	13 (56.5)	5 (21.7)	2 (8.7)	3 (13.0)
Human	23 (46.0)	10 (20.0)	6 (12.0)	11 (22.0)
Chicken	22 (43.1)	11 (21.7)	10 (19.6)	8 (15.7)
Cattle	43 (84.3)	7 (13.7)	1 (2.0)	0 (0)
Total	101 (57.7)	33 (18.9)	19 (10.9)	22 (12.6)

a Penicillins, monobactams, third-generation cephalosporins, tetracyclines, phenicols, and quinolones.

b Resistance to 3 or more antibiotic categories was classified as multidrug resistant.

Resistance was more commonly observed in E. coli isolated from chickens (56.9%) and humans (54.0%) than in E. coli from ruminants (15.7%). The proportion of E. coli isolates from soil resistant to at least one antibiotic category (43.5%) was closer to the proportional resistance among E. coli from chicken and human isolates than in isolates from ruminants. Notably, 13.0% of soil isolates were MDR (Table 2). The similarities in prevalence and resistance patterns observed among E. coli isolates from soils, human feces, and chicken feces align with prior work identifying similar genotypic and phenotypic characteristics among isolates from soil, human feces, and chicken feces (40). The data support the potential for human and/or chicken feces to be a source of soil E. coli. Although antibiotic resistance data of E. coli from household soils is scarce, as the majority of prior studies focused on resistance in agricultural soils, the prevalence observed here is concerning, especially considering that we did not use antibiotic-selective media for isolation. Whether the E. coli strains isolated are a result of direct fecal input or if they represent environmental populations that are genetically different from the fecal sources is currently unknown and represents an interesting research topic for further investigation.

Interestingly, resistance to third-generation cephalosporins was detected with a frequency slightly higher in E. coli isolated from soils than in E. coli isolated from fecal sources (Table S3). Third-generation cephalosporins are an important family of antibiotics widely used for the treatment of infections with Gram-negative bacteria. Soils are regarded as selective environments due to the presence of many antibiotic compounds produced by soil bacteria (41). Furthermore, anthropogenic release of antibiotics and antibiotic derivatives into soils may contribute to the proliferation of antibiotic-resistant bacteria (18). For example, most cephalosporins administered parenterally to humans and animals are eliminated rapidly through urine (42). Therefore, the selection of antibiotic-resistant bacteria not only occurs in the individual or animal taking the antibiotic but may also occur in the environmental compartment receiving the residues (18). Nonetheless, it is important to consider that soil resistomes are complex, and antibiotic resistance genes have been documented in high abundance in soils regardless of recent anthropogenic influence (43, 44).

We found 10 isolates to be resistant to third-generation cephalosporins, of which seven (two isolated from soils) were confirmed to be extended-spectrum beta lactamase (ESBL) producers by the double-disk synergy test (DDST) and carried the beta-lactamase gene *bla*_CTX-M-group-1_. In addition, two isolates coharbored another ESBL gene (*bla*_TEM_ or *bla*_OXA-1-like_). The presence of E. coli resistant to third-generation cephalosporins (including ESBL producers) in domestic soils in Bangladesh suggests that this environmental compartment may play a role in child exposures to antimicrobial-resistant bacteria. Children (3 to 18 months) in a similar setting were observed to frequently ingest soil and to mouth hands and objects after touching soil (8, 15). Exposure to ESBL-producing organisms through soil contact is concerning, as septicemia caused by ESBL-producing organisms has an elevated risk for fatality relative to septicemia caused by antibiotic-susceptible infections (45).

### Distribution of intestinal pathotypes among E. coli from soil and fecal sources.

Overall, 10.3% of the 175 E. coli isolates possessed at least one of 10 intestinal virulence-associated genes tested. Enteropathogenic E. coli (EPEC) was the most prevalent pathotype encountered (4.6%), with seven of the eight EPEC isolates classified as atypical EPEC (only carrying the *eae* gene) and the other as typical EPEC (carrying both *eae* and *bfp* genes). EPEC was more frequently found in E. coli isolated from chicken feces (7.8%) than other sources, and it was the only pathotype detected in chicken feces. In contrast, Shiga toxin-producing E. coli (STEC) marked by the presence of *stx*_1_ or *stx*_2_ was only detected in cattle feces. Of the 51 cattle isolates tested, 11.8% were classified as STEC. Human fecal isolates showed a higher diversity of virulence-associated genes (*eae*, *bfp*, *aaiC*, and *lt*), as three different pathotypes (EPEC, enteroaggregative E. coli [EAEC], and enterotoxigenic E. coli [ETEC]) were detected in human fecal isolates. From soil samples, one isolate was found to carry *aat* and *aaiC*, indicative of EAEC, while another isolate carried *eae*, indicative of atypical EPEC (Table 3). The detection of *eae* gene in DNA extracted directly from soils revealed the presence of EPEC in an additional soil sample. Enteroinvasive E. coli (EIEC) was not detected in any of the studied isolates (Table 3).

**TABLE 3:** Distribution of intestinal pathotypes of *E. coli* isolated from soil and fecal samples

Source	No. of E. coli strains	No. (%) of isolates positive for intestinal pathogenic virulence-associated genes					
		EAEC^*a*^	EIEC^*b*^	EPEC^*c*^	ETEC^*d*^	STEC^*e*^	Any IPEC
Soil	23	1 (4.4)	0 (0)	1 (4.4)	0 (0)	0 (0)	2 (8.7)
Human	50	2 (4.0)	0 (0)	2 (4.0)	1 (2.0)	0 (0)	5 (10.0)
Chicken	51	0 (0)	0 (0)	4 (7.8)	0 (0)	0 (0)	4 (7.8)
Cattle	51	0 (0)	0 (0)	1 (2.0)	0 (0)	6 (11.8)	7 (13.7)
Total	175	3 (1.7)	0 (0)	8 (4.6)	1 (0.6)	6 (3.4)	18 (10.3)

a EAEC, indicated by the presence of *aat* or *aat* and *aaiC*.

b EIEC, genes *ial* and *ipaH* were not detected.

c EPEC, indicated by the presence of *eae* or *eae* and *bfp*.

d ETEC, indicated by the presence of *lt*.

e STEC, indicated by the presence of *stx*_1_ or *stx*_1_ and *stx*_2_.

Notably, the proportion of potentially pathogenic E. coli reported in this study is not directly comparable to the proportions in other studies where enrichment for pathotypes or pooled DNA extraction followed by molecular methods were performed (17, 46). In our study, E. coli was isolated in tryptone bile X-glucuronide (TBX) agar, which is a selective agar for E. coli detection irrespective of pathogenicity; thus, the E. coli isolated in this medium represents the total culturable E. coli present in the samples. The presence of virulence genes in 8.7% of the randomly selected E. coli colonies recovered from soil samples (one per sample) suggests that within this study site, a surprisingly high proportion of E. coli strains in soil are potentially pathogenic.

### Survival and growth of EPEC in domestic soil microcosms.

**FIG 1:**
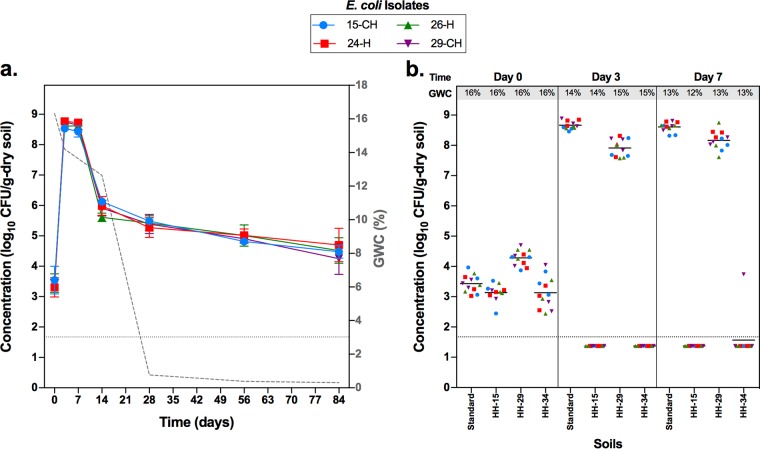
Survival dynamics of four EPEC isolates (15-CH, 24-H, 26-H, and 29-CH) in autoclaved soils. (a) Geometric mean log_10_ CFU per gram of dry soil of four EPEC isolates measured at days 0, 3, 7, 14, 28, 56, and 84 after spiking standard soil. Each symbol represents the geometric mean log_10_ CFU per gram of dry soil, and the error bar indicates the standard deviation of three independent replicates per isolate. The lower limit of detection (LOD) is indicated by the horizontal dotted line. Gravimetric water content (GWC) of the soil at each time point is indicated by the dotted line and the right *y* axis. (b) Aggregate of the concentration of four EPEC isolates in the standard soil and soils collected from three households (HH-15, HH-29, and HH-34). Each symbol represents the log_10_ CFU per gram of dry soil for each isolate and their replicates (three independent replicates per isolate); the horizontal line is the geometric mean log_10_ CFU per gram of dry soil of all the isolates for each soil type (GWC is indicated) and on each sampling day (days 0, 3, and 7), and the dotted line indicates the lower LOD. When the CFU counts were below the lower LOD, the value used to graph corresponds to half the lower LOD.

Four EPEC isolates, including both antibiotic-sensitive and -resistant strains (Table S4), readily grew in the autoclaved natural standard soil, a commercially available sandy loam soil described further in Materials and Methods. Specifically, substantial growth was observed from day 0 (seeded at a concentration of ∼10^3^ CFU/g dry soil) to day 3, when all isolates were detected at concentrations of 10^8^ CFU/g dry soil (Fig. 1a). Beyond day 3, the concentration decreased but remained higher than the concentrations observed immediately after spiking (Fig. 1a). The kinetics of growth and persistence were similar for all four isolates (Fig. 1a and S2a). In contrast, in nonautoclaved soil, there was a sharp decrease in concentration at day 7 postseeding (Fig. S2b). By day 14, all four isolates were no longer detectable. These results support previous findings that soil microflora reduce the survival of E. coli in soil environments (28, 29). Soil microflora impacts E. coli survival and/or growth through competition for available nutrients and/or a direct antagonistic relationships, such as predation by protozoa (47–49). Additionally, autoclaving the soil may promote E. coli growth through release of nutrients, as, for example, ammonium-N (50). In addition, the availability of organic compounds is important for E. coli growth in soil environments (23, 27). Interestingly, adapting the EPEC isolates in autoclaved soil before facing nonautoclaved soil substantially extended the survival time (Fig. S2c). The adaptation experiment here is analogous to E. coli entering the environment via feces.

**FIG 2:**
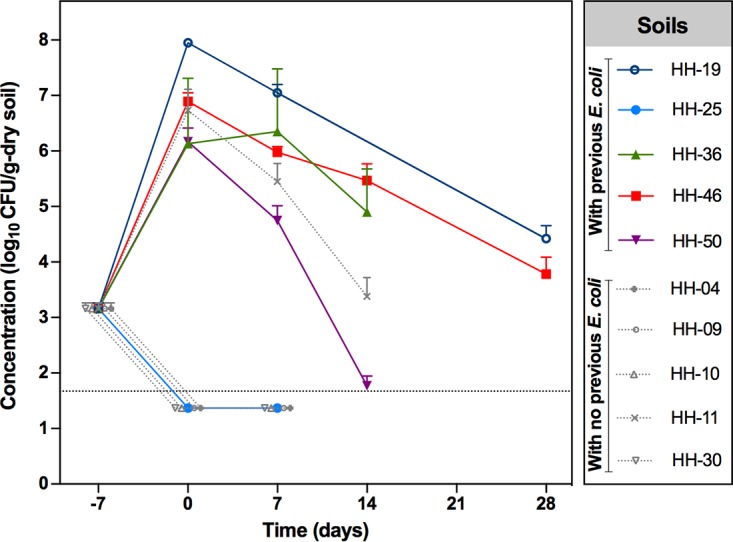
Survival dynamics of *E. coli* 26-H (isolated from human feces, classified as typical EPEC, resistant to third-generation cephalosporins, ESBL producer, and carrier of the CTX-M beta-lactamase) in 10 Bangladeshi household soils. Each symbol represents the geometric mean log_10_ CFU per gram of dry soil, and the error bar indicates the standard deviation of three independent replicates per soil at days 0, 7, 14, and 28 (only for two soil). Day −7 represents the calculated CFU per gram used to seed the autoclaved fraction of the soils; day 0 is the CFU per gram of dry soil after spiking the nonautoclaved portion with the seeded autoclaved soil (1:19 autoclaved to nonautoclaved ratio). The dotted line indicates the lower limit of detection (LOD). When the CFU counts were below the lower LOD, the value used to graph corresponds to half the lower LOD.

We next followed the fate of the four EPEC isolates in three other soils collected from the households (soils HH-15, HH-29, and HH-34; Table S5). While no significant growth or survival differences were seen among the four isolates, we observed that growth varied by soil source (Fig. 1b). While the concentrations of the isolates increased in one soil (soil HH-29), mirroring what was observed in the natural standard soil, concentrations of all isolates fell below the detection limit as early as day 3 postseeding in the other two soils (soils HH-15 and HH-34) (Fig. 1b). These striking differences in EPEC growth and survival among different soils collected from the households led us to study more Bangladeshi soils. In total, we selected 10 soils, five of which had detectable E. coli and five of which did not at the time of sampling in the households (Table S5). Growth and survival kinetics of one E. coli strain (26-H; isolated from human feces, classified as typical EPEC, resistant to third-generation cephalosporins, is an ESBL producer, and carries a CTX-M group 1 beta-lactamase; Table S4) was observed in half of the soils (Fig. 2). Specifically, in four of the five soils where E. coli was detected at the time of collection, isolate 26-H was able to persist for 14 days after spiking the nonautoclaved soil fraction. In the other soil (soil HH-25), isolate 26-H did not grow or persist (Fig. 2). In contrast, in four of the five soils with no previous E. coli detection, isolate 26-H was not detected after spiking the nonautoclaved soil. One soil (soil HH-11) with no previous E. coli detection was permissive of E. coli survival (Fig. 2). No obvious soil characteristic related to growth was identified. For example, the pH values of the 10 soils tested were very similar and close to neutral values (Table S5). Furthermore, soil-derived supernatant did not directly inhibit E. coli growth on laboratory media, suggesting that no E. coli growth inhibitor is present in the soils (data not shown).

E. coli growth is dependent on soil moisture content, as observed in soil microcosms. Specifically, E. coli 26-H rapidly decreased in number below the lower limit of detection in autoclaved soil 2.2, with an adjusted moisture content of 5% (field capacity, ∼44.8%) (Fig. S3). In contrast, when the moisture content of soil 2.2 was adjusted to 10%, 15%, or 20%, the concentration of the isolate increased by 5 orders of magnitude (from ∼10^3^ to ∼10^8^ CFU/g [dry soil weight]) within 7 days. The results align with the aforementioned observed correlation between soil moisture content and E. coli concentrations. The results also align with prior work identifying water content as a major driver of survival kinetics of bacteria in soils (27, 28, 51), especially at growth-permissive temperatures. Notably, small differences in soil moisture content may also influence E. coli survival and/or growth, particularly in the presence of soil microflora, which contributes to a more competitive environment. Quantification using culture-based methods may also influence recovery, as they may be unable to recover stressed bacterial cells, as, for example, cells that have entered the viable but nonculturable (VBNC) state, at low moisture content, or under other environmental stressors (52, 53).

Overall, the findings of our study indicate that soil physicochemical properties influence the detectability, concentration, and growth potential of E. coli (including potentially pathogenic and antibiotic-resistant variants) in households in rural Bangladesh. In contrast, WASH indicators were not significantly associated with E. coli contamination of household soils in our study site. These findings suggest that studies investigating the transmission of E. coli in household environments should consider soil ecology to be a moderating variable between household-level risk factors and E. coli detection. Soils may act as reservoirs in E. coli transmission by enabling the growth of antibiotic-resistant and potentially pathogenic E. coli variants, as demonstrated by our microcosm studies. Risks from E. coli growth in soil are high, given the observed high rates of soil ingestion (both directly and indirectly) among children in Bangladesh and other LMICs. We also found that strain-specific adaptations to growth in soil may not be compulsory for persistence in soil, as no differences in growth and survival rates among the isolates were observed. Moreover, the presence and demonstrated growth of pathogenic and antimicrobial-resistant E. coli strains in these household soils suggest that other pathogenic bacterial species with ecology similar to E. coli may have the potential to persist and/or grow in soil and therefore also pose a risk to human health.

Further studies are warranted to determine the importance of growth and persistence of E. coli and other pathogens *in situ* to complement our microcosm evidence. Elucidating the origin and fate of pathogenic bacteria in domestic soil environments is important in order to design effective measures to control transmission. For example, programs to promote upgrading soil flooring in households may help reduce pathogen transmission, as shown by the 13% reduction in diarrheal disease observed in Mexico’s Piso Firmo program (54).

## MATERIALS AND METHODS

### Ethics statement and study site.

This study was performed following an approved protocol by the ethics committees of the Swiss Federal Institute of Technology Zurich (ETH Zurich, Switzerland) and the International Centre for Diarrhoeal Disease Research, Bangladesh (icddr,b; Dhaka, Bangladesh). The study was conducted in 52 households with dirt/soil flooring located in rural villages of Mirzapur Upazila in the Tangail district of Bangladesh (26 households with ruminants and 26 households without ruminants) during February to April 2016. Researchers/enumerators from icddr,b conducted household surveys, soil sampling, and fecal sampling. A questionnaire-based survey was conducted on household assets and infrastructure, gastrointestinal/respiratory illness among household members, and agricultural/livestock practices, as well as spot-check observations for WASH infrastructure. Based on household assets, infrastructure, and livestock ownership, household wealth was indicated by constructing a composite wealth index using principal-component analysis and k-means clustering. Environmental and fecal sampling included the collection of one soil sample, one human fecal sample, one chicken fecal sample, and one cattle (ruminant) fecal sample (if present) from each participating household, as later described.

### Soil and fecal sample collection.

Soil samples (*n* = 52) from the front yard of the households with no visible feces, food or trash, were collected. Approximately 150 g of soil was aseptically retrieved from an area of 60 cm^2^ and <2 cm depth, and stored on ice in a sterile Fisherbrand sample bag (Fisher Scientific, PA, USA). Human fecal samples were provided in a stool container by household members (18 to 64 years old). Fecal samples from chickens and cattle, with fresh and glossy appearance, preferably right after observing the animal deposit the feces, were aseptically collected by the enumerator. Samples were stored on ice and transported to the Enteric and Food Microbiology Laboratory at icddr,b, where they were stored at 4°C and processed within 24 h of collection.

### Soil physicochemical analyses.

Soil physicochemical analyses were performed at the Department of Soil, Water and Environment of the University of Dhaka. For all the soil samples, soil dry gravimetric water content (GWC) was determined by drying 1 g of soil at 100°C for 16 h or until mass remained constant. Field capacity (55) and permanganate oxidizable active organic carbon (in milligrams per kilogram) (56, 57) were also measured. In addition, for a subset of 30 soil samples, particle size (58), active organic carbon (%C) with the Walkley-Black chromic acid wet oxidation method (59), and total nitrogen (%N) by the Kjeldahl method (60) were determined. For 10 soils used for the microcosm studies, soil pH was determined in a 0.01 M calcium chloride solution at 1:1 soil-solution ratio (61).

### E. coli enumeration and isolation.

E. coli enumeration and isolation from soil and fecal samples were performed as previously described (33), with slight modifications. In brief, 5 ± 0.25 g of soil or 1 ± 0.25 g of feces was diluted in a sterile Fisherbrand blender bag (Fisher Scientific, PA, USA) and mixed by hand for 2 min in 30 ml of phosphate-buffered saline (PBS). The mixture was left to settle for 15 ± 3 min to allow sedimentation of bigger particles and for reproducible 10-fold serial dilutions. For enumeration of E. coli from soils, 1 ml of each dilution was inoculated onto tryptone bile X-glucuronide (TBX) agar (Oxoid, Basingstoke, UK) by the pour plate technique. The lower limit of detection (LOD) for the soil samples was 0.99 log_10_ CFU/g of dry soil. For isolation of E. coli from feces, 100 μl of each dilution was spread plated onto TBX agar. All plates were incubated at 37°C for 18 to 24 h, and one colony (for soil, human, and chicken samples) or two colonies (for cattle samples) were selected, based on blue-green color appearance on the TBX medium, for species confirmation using the API-20E system (bioMérieux, Marcy-L’Étoile, France). The confirmed E. coli isolates were given a number corresponding to the household where the sample was collected (1 to 52), followed by the sample type, as follows: “S” for soil, “H” for human fecal, “CH” for chicken fecal, and “C” for cattle fecal (i.e., 15-CH corresponds to the E. coli isolate recovered from a chicken fecal sample collected from household 15). E. coli isolates were stored at −80°C at the icddr,b and sent to Eawag (Dübendorf, Switzerland) for further analyses.

### Random amplified polymorphic DNA.

RAPD fingerprinting was performed on E. coli strains isolated from soils, using primer “4” (5′-AAGAGCCCGT-3′) (discrimination index, 0.983) and following a procedure described previously (62). Results were analyzed with the software BioNumerics 4.5. Similarity was determined using the Dice coefficient, and clustering was performed by the unweighted pair group method with arithmetic means (UPGMA). RAPD patterns with a Dice coefficient of >80% were considered to be probably related and assigned to the same cluster or RAPD type.

### Molecular detection of E. coli in soils.

Molecular detection targeting the conserved beta-glucuronidase gene *uidA* in DNA extracted from soils was performed to establish whether or not the culture-based approach resulted in false negatives. For DNA isolation from soil, 0.25 g of soil was additionally collected from each household and added to a cryovial containing 1 ml of LifeGuard soil preservation solution (Qiagen, Hilden, Germany). Soil samples were stored at −20°C and processed before 30 days after collection. DNA was extracted using the PowerSoil DNA isolation kit (Mo Bio, CA, USA), following the manufacturer’s instructions. Molecular detection of E. coli was performed by PCR, using primers targeting the beta-glucuronidase gene *uidA* (*uidA*_For, 5′-GCGTCTGTTGACTGGCAGGTGGTGG-3′; and *uidA*_Rev, 5′-GTTGCCCGCTTCGAAACCAATGCCT-3′), a gene commonly found in E. coli (63) Reaction conditions were as follows: 95°C for 5 min, followed by 30 cycles of 95°C for 30 s, 63°C for 30 s and 72°C for 30 s, and a final extension at 72°C for 5 min. DNA extracted from the E. coli strain ATCC 25922 was used as positive control, while DNase-free water was used as a nontemplate control.

### Antibiotic susceptibility testing.

The antibiotic susceptibilities of the 175 isolated E. coli strains was determined against 16 different antibiotic disks (Oxoid, Basingstoke, UK) by standard disk diffusion technique, following the Clinical and Laboratory Standards Institute (CLSI) guidelines and interpretation standards (64). The evaluated antibiotics included representatives of five different antibiotic categories, as follows: the beta-lactams ampicillin (AMP) and amdinocillin (MEC) (penicillins); piperacillin-tazobactam (TZP) (beta-lactam–beta-lactam inhibitors); aztreonam (ATM) (monobactam); cefixime (CFM), ceftriaxone (CRO), cefotaxime (CTX), and ceftazidime (CAZ) (third-generation cephalosporins); meropenem (MEM) and imipenem (IPM) (carbapenems); the aminoglycoside amikacin (AMK); tetracycline (TET); the phenicol chloramphenicol (CAM); the quinolones nalidixic acid (NAL) and ciprofloxacin (CIP); and the folate pathway drug trimethoprim-sulfamethoxazole (SXT). Multidrug resistance was defined as nonsusceptibility to at least one antibiotic in 3 or more categories as defined by Magiorakos et al. (65). Double-disk synergy test (DDST) was carried out on 10 E. coli isolates suspected to be ESBL producers (based on their resistance to third-generation cephalosporins). The DDST was considered positive when expansion of the inhibition zone of CTX, CRO, and/or ATM disks toward a disk with clavulanic acid located 20 mm away was observed, as indicated by Jalier et al. (66), with some modifications (67).

### Detection of virulence-associated and extended-spectrum beta-lactamases-encoding genes by PCR.

Previously described PCR methods (68) were used for the detection of the following 10 virulence-associated genes indicative of five different E. coli intestinal pathotypes in the 175 E. coli isolates: enteroaggregative E. coli (EAEC) genes *aaiC* (secreted protein) and *aat* (antiaggregation protein transporter gene); enteroinvasive E. coli (EIEC) genes *ial* (invasion associated locus) and *ipaH* (invasion plasmid antigen H); enteropathogenic E. coli (EPEC) genes *eae* (intimin) and *bfp* (bundle-forming pilus); enterotoxigenic E. coli (ETEC) genes *lt* (heat labile enterotoxin) and *st* (heat stable enterotoxin), and Shiga toxin-producing E. coli (STEC) genes *stx*_1_ and *stx_2_* (Shiga toxins). Detection of *eae* was also directly performed on the DNA isolated from soils. In addition, detection of the beta-lactamase genes *bla*_TEM_, *bla*_SHV_, *bla*_OXA-1-like_, and *bla*_CTX-M_, was performed on all ESBL-producing E. coli isolates by multiplex PCR with previously described primers (69). A bacterial strain known to carry the gene targeted by each primer pair was used as a positive control. E. coli strain ATCC 25922 and water were used as negative and nontemplate controls, respectively.

### Soil microcosm studies.

Growth and survival in soil were evaluated for four EPEC isolates (15-CH, 24-H, 26-H, and 29-CH), including one isolate sensitive to all antibiotics, while the other three isolates showed different resistance profiles (Table S4). Some experiments were conducted only with the EPEC isolate 26-H (resistant to third-generation cephalosporins, an ESBL producer, and a carrier of the CTX-M beta-lactamase). Experiments were performed with a natural standard soil (soil type no. 2.2) from LUFA Speyer Germany (http://www.lufa-speyer.de/index.php) and 13 soils collected from the households. The natural standard soil no. 2.2 is a commercially available sandy loam soil with known physicochemical properties (Table S5) and has not received pesticides, biocidal fertilizers, or organic manure for at least 5 years; therefore, it was used here as a control soil. Soils were sieved through a 2.36-mm mesh followed by sterilization by 3 consecutive rounds of autoclaving. Soil GWC was determined with 0.5 g of soil following the procedure mentioned earlier. Before starting the experiments, the soil GWC was adjusted to 15% ± 1% GWC with sterile double-distilled water (ddH_2_O). For one experiment, the soil GWC was adjusted only at the start of the experiment (Fig. 1), while for the others (Fig. 2, S2, and S3), the GWC was adjusted if necessary after each time point measured. As autoclaving the soil impacts the indigenous soil microbiota and likely affects some physicochemical soil properties (70, 71), we compared the survival dynamics of the four EPEC isolates in autoclaved versus nonautoclaved standard soil. To find a scenario that likely resemble a more realistic condition that E. coli encounters in domestic soil, a mix of sterilized and unsterilized soil in a ratio of 1:19 or 1:1 was used. In this case, the sterile autoclaved soil fraction was seeded with E. coli and incubated for 7 days before spiking the nonautoclaved soil fraction with the seeded autoclaved soil. The GWC-adjusted soils (4 to 5 g) were placed into 50-ml tubes and maintained at room temperature until used. Triplicate soil samples were prepared for each condition and for each E. coli isolate evaluated. For inoculation into the soils, E. coli cells were prepared as previously described, with modifications (72). In brief, each E. coli isolate from overnight cultures in LB broth was diluted into the same medium in triplicates to a starting OD_600_ of 0.05 and grown to mid-logarithmic phase (OD_600_, 0.6) at 37°C and 220 rpm. Cells were harvested at 6,500 × *g* for 5 min, washed twice with 1× PBS to avoid media carryover, and resuspended in 1× PBS to an estimated 10^8^ CFU/ml. The cell suspension was diluted, and soils were inoculated to a concentration of 10^1^ to 10^4^ CFU/g of dry soil. As noninoculated and water content controls, sterile ddH_2_O was added instead of the bacterial suspension. Soil-bacterium microcosms were mixed by inversion for 1 min, followed by vortexing at maximum speed for 1 min. Right after mixing (day 0), the CFU per gram of dry soil was measured by withdrawing and suspending approximately 0.5 g of the inoculated soil (exact weights were recorded for each sample) into 1× PBS, followed by 1 min of vortexing at maximum speed and centrifugation at low speed (200 × *g* for 2 min) to sediment soil particles. The resulting supernatant was subjected to 10-fold serial dilutions, and a 25-μl volume from each dilution was drop-plated in duplicate in TBX agar (73). The number of CFU was counted after overnight incubation at 37°C. The microcosms were incubated at 30°C, which is within the range of average temperature in the study area. Furthermore, Islam et al. reported significant linearity between atmospheric temperature and soil temperature at 5 cm depth in Bangladesh (74). Bacterial counts were determined at different time points over a period of up to 84 days, as described for day 0. The lower LOD for each microcosm experiment is indicated in the corresponding graphs.

### Inhibition assay.

Inhibitory effect of soil on growth of E. coli was investigated with six Bangladeshi soils, three that were positive for E. coli isolation (HH-25, HH-46, and HH-50) and three negative for E. coli isolation (HH-04, HH-09, and HH-10). For this, a 1:1 soil-to-PBS solution was prepared, vortexed at maximum speed for 1 min, and centrifuged at 200 × *g* for 2 min. Ten microliters of the supernatant from each soil-PBS solution was applied to the center of a Mueller-Hinton agar plate previously inoculated with the E. coli strain ATCC 25922. Zones of inhibition were measured after overnight incubation at 37°C.

### Statistical analyses.

Data were analyzed using GraphPad Prism, version 7.0a (GraphPad Software, Inc., La Jolla, CA) and R version 3.4.3. All concentrations are expressed as log_10_ E. coli CFU per gram of dry soil, as the soil GWC was determined. When the CFU counts were below the lower LOD, half the lower LOD was assumed for all subsequent quantitative analyses. Wilcoxon signed rank test and Kruskal-Wallis test by ranks were used to compare mean ranks of E. coli concentrations in soil among groups obtained from the survey data. To evaluate if the presence of ruminants is associated with presence and concentration of E. coli in soils, Fisher’s exact and Wilcoxon signed rank test were used, respectively. The association between log_10_ E. coli CFU/g of dry soil and monthly expenditures, toilet age, or the soil physicochemical parameters was evaluated using Spearman’s rank correlation analysis. Differences in the proportion of resistant isolates among sources were evaluated using Fisher’s exact. For the soil microcosm results, significant differences in the geometric mean of log_10_ E. coli CFU/g of dry soil were evaluated using one-way analysis of variance (ANOVA) with *post hoc* analysis (Tukey’s multiple-comparison test) or independent Student’s *t* test.

## Supplementary Material

Supplemental file 1
