# Supplementary material for: Risk Factors for Detection, Survival, and Growth of Antibiotic-Resistant and Pathogenic Escherichia coli in Household Soils in Rural Bangladesh
Source: Appl Environ Microbiol. 2018 Nov 30;84(24):e01978-18. doi: 10.1128/AEM.01978-18 (PMC6275341; doi:10.1128/AEM.01978-18)
Supplement: Supplemental file 1 [file zam024188893s1.pdf]

SUPPLEMENTAL MATERIAL

Risk factors for detection, survival, and growth of antibiotic-resistant and pathogenic *Escherichia coli* in household soils in rural Bangladesh

Maria Camila Montealegre, Subarna Roy, Franziska Böni, Muhammed Iqbal Hossain, Tala Navab-Daneshmand, Lea Caduff, A.S.G. Faruque, Mohammad Aminul Islam, Timothy R. Julian.

Table of Contents

Table S1. Monthly expenditures, durable assets, household characteristics, and livestock ownership among study households, used to construct wealth indices, as compared to 2014 Demographic and Health Survey (DHS) for Rural Bangladesh.

Table S2. Physicochemical characteristics measured among the soils collected from households in Mirzapur, Bangladesh and correlation coefficient with *E. coli* concentration in soils.

Table S3. Distribution of *E. coli* isolates by source and resistance to 16 different antibiotics.

Table S4. Characteristics of the four *E. coli* isolates used in microcosm experiments.

Table S5. Physicochemical characteristics of the standard soil No. 2.2 from LUFA Speyer Germany and 13 domestic soils collected from households of Mirzapur, Bangladesh used in microcosm experiments

Figure S1. RAPD fingerprinting of 23 *E. coli* isolated from household soils of Mirzapur, Bangladesh.

Figure S2. Survival dynamics of four *E. coli* isolates (15-CH, 24-H, 26-H, and 29-CH) in **(a)** autoclaved, **(b)** non-autoclaved and **(c)** a 1:1 ratio of autoclaved: non-autoclaved standard soil.

Figure S3. Survival dynamics of *E. coli* 26-H (isolated from human feces, classified as typical EPEC, resistant to third-generation cephalosporins, ESBL producer, and carrier of the CTX-M beta-lactamase) in autoclaved standard soil under different moisture conditions.

Table S1. Monthly expenditures, durable assets, household characteristics, and livestock ownership among study households, used to construct wealth indices, as compared to 2014 Demographic and Health Survey (DHS) for Rural Bangladesh

	2014 DHS (Rural Bangladesh)	This Study	PCA[a] Loading for Wealth Indices
**Monthly Expenditure (taka)**			
Mean ± SD		9578 ± 4788	
Median		9000	
**Durable Assets**			
Electricity	0.50	0.76	**0.71**
Television	0.32	0.50	**0.71**
Mobile Telephone	0.87	0.96	*excluded*
Refrigerator	0.11	0.25	**0.71**
Wardrobe	0.37	0.06	0.37
Electric Fan	0.48	0.71	**0.69**
Computer/Laptop	0.02	0.02	*excluded*
Rickshaw/Van	0.06	0.17	-0.15
Bicycle	0.3	0.36	0.21
**Household Characteristics**			
Ownership	0.94	0.96	*excluded*
Flooring			
Earth[b]	0.83	1.00	
Walls			
Tin	0.45	0.98	*excluded*
Cement	0.16	0.02	*excluded*
Other	0.39	0.00	
Roof			
Tin	0.9	1.00	*excluded*
Other	0.1	0.00	
Latrine			
Improved - Basic	0.44	0.71	**0.44**
Improved - Limited	0.33	0.25	-0.39
Unimproved	0.19	0.04	*excluded*
**Livestock**			
Buffalo			
None	0.995	1.00	*excluded*
>1	0.005	0	
Cows/Bulls			
None[b]	0.58	0.5	
1-4	0.38	0.49	**0.42**
>5	0.04	0.04	0.25
Goat/Sheep			
None	0.76	0.96	
1-4	0.22	0.04	**0.42**
>5	0.03	0	*excluded*
Chickens/Ducks			
None	0.31	0	
1-9	0.47	0.71	*excluded*
>10	0.22	0.29	*excluded*
Other Farm Animals			
None	0.95	1	

[a] PCA refers to principle components analysis used to define wealth quartiles, loadings > 0.40 are emboldened.
[b] Study inclusion criteria included earth floors for all households and cow/bull ownership in half (n = 26) of households.

Table S2. Physicochemical characteristics measured among the soils collected from households in Mirzapur, Bangladesh and correlation coefficient with *E. coli* concentration in soils

	Soil characteristics							
						Particle size (mm)[b]		
	Moisture content (%)	Field capacity (%)	POxC[a] (mg/kg)	Active organic carbon (%C)	Total nitrogen (%N)	Clay (<0.002)	Silt (0.002-0.05)	Sand (0.05-2)
**n[c]**	52	52	52	29	37	30	30	30
**Mean**	20.82	34.79	252.2	0.65	0.10	22.73	49.63	27.63
**SD**	6.96	7.52	151.8	0.46	0.05	7.39	10.35	12.6
**ρ[d]**	0.48	-0.23	-0.19	-0.28	-0.11	-0.47	-0.07	0.29
**P**	**0.0003**	0.0973	0.1711	0.1392	0.5089	**0.0095**	0.7222	0.1234

[a]Permanganate oxidizable active organic carbon.

[b]Particle size (mm) distribution according to USDA (%).

[c]Number of soils measured.

[d]Spearman's rank correlation coefficient.

Table S3. Distribution of *E. coli* isolates by source and resistance to 16 different antibiotics

Antibiotic[a]	No. (%) of resistant isolates by source				
	Soil n=23	Human n=50	Chicken n=51	Cattle n=51	Total n=175
B-lactams					
AMP	6 (26.1)	13 (26.0)	15 (29.4)	2 (3.9)	36 (20.6)
MEC	1 (4.4)	3 (6.0)	1 (2.0)	2 (3.9)	7 (4.0)
TZP	0 (0)	0 (0)	0 (0)	0 (0)	0 (0)
ATM	2 (8.7)	2 (4.0)	2 (3.9)	0 (0)	6 (3.4)
CAZ	1 (4.4)	1 (2.0)	0 (0)	0 (0)	2 (1.1)
CTX	2 (8.7)	3 (6.0)	2 (3.9)	0 (0)	7 (4.0)
CRO	2 (8.7)	3 (6.0)	2 (3.9)	0 (0)	7 (4.0)
CFM	3 (13.0)	5 (10.0)	2 (3.9)	0 (0)	10 (5.7)
MEM	0 (0)	0 (0)	0 (0)	0 (0)	0 (0)
IPM	0 (0)	0 (0)	0 (0)	0 (0)	0 (0)
Aminoglycoside					
AMK	0 (0)	0 (0)	0 (0)	0 (0)	0 (0)
Tetracycline					
TET	7 (30.4)	16 (32.0)	22 (43.1)	3 (5.9)	48 (27.4)
Phenicols					
CAM	1 (4.4)	1 (2.0)	2 (3.9)	0 (0)	4 (2.3)
Quinolones					
NAL	2 (8.7)	12 (24.0)	7 (13.7)	1 (2.0)	22 (12.6)
CIP	2 (8.7)	5 (10.0)	1 (2.0)	0 (0)	8 (4.6)
Folate pathway					
SXT	2 (8.7)	8 (16.0)	7 (13.7)	1 (2.0)	18 (10.3)

[a]AMP, ampicillin; MEC, mecillinam; TZP, piperacillin-tazobactam; ATM, aztreonam; CFM, cefixime; CRO, ceftriaxone; CTX, cefotaxime; CAZ, ceftazidime; MEM, meropenem; IPM, imipenem; AMK, amikacin; TET, tetracycline; CAM, chloramphenicol; NAL, nalidixic acid; CIP, ciprofloxacin; SXT, trimethoprim-sulfamethoxazole.

Table S4. Characteristics of the four *E. coli* isolates used in microcosm experiments

E. coli isolate	Source	Intestinal virulence gene	Antibiotic[a] resistance profile	ESBL[b] gene
15-CH	Chicken	*eae*	AMP, MEC	ND[c]
24-H	Human	*eae*	ND[c]	ND[c]
26-H	Human	*eae, bfp*	AMP, CFM CRO, CTX	$bla_{\text{CTX-M-group-1}}$
29-CH	Chicken	*eae*	TET	ND[c]

[a]AMP, ampicillin; MEC, mecillinam; CFM, cefixime; CRO, ceftriaxone; CTX, cefotaxime; TET, tetracycline.

[b]Extended-spectrum beta-lactamase.

[c]Not detected.

Table S5. Physicochemical characteristics of the standard soil No. 2.2 from LUFA Speyer Germany and 13 domestic soils collected from households of Mirzapur, Bangladesh used in microcosm experiments

Soil	Moisture content (%)	Field capacity (%)	POxC[a] (mg/kg)	Active organic carbon (%C)	Total Nitrogen (%N)	pH	Particle size (mm)[b]			Soil type	*E. coli* (CFU/g dry soil)	Growth in soil microcosm
							Clay (<0.002)	Silt (0.002-0.05)	Sand (0.05-2)			
**Standard 2.2**	NA[c]	44.8±2.9	ND[d]	1.61±0.15	0.17±0.01	5.4±0.2	8.0±1.7	15.8±3.1	76.2±4.1	Sandy loam	NA[c]	Yes
**HH-04**	16.09	59.43	124.488	ND[d]	0.0748	6.31	45	49	6	Silty clay	4.9[e]	No
**HH-09**	20.48	33.27	76.61	0.29	0.074	6.78	16	48	36	Loam	4.9[e]	No
**HH-10**	20	42.54	181.94	0.49	0.0742	6.89	19	61	20	Silt loam	4.9[e]	No
**HH-11**	16.09	34.23	129.28	0.52	0.1341	6.54	24	56	20	Silt loam	4.9[e]	Yes
**HH-15**	27.85	40.26	632.02	1.42	0.1963	ND	29	58	13	Silty clay loam	4.9[e]	No
**HH-19**	34.67	31.59	177.16	0.49	0.09	7.02	16	63	21	Silt loam	1.4x10^3	Yes
**HH-25**	31.71	36.12	231.62	ND[d]	0.112	6.46	ND[d]	ND[d]	ND[d]	ND[d]	1.9x10^2	No
**HH-29**	19.04	31.09	172.37	0.19	0.0788	ND	14	41	45	Loam	2.6x10^2	Yes
**HH-30**	22.89	27.81	67.03	0.23	0.0723	6.34	29	36	35	Clay loam	4.9[e]	No
**HH-34**	30.77	38.45	129.276	0.52	0.0781	ND	32	47	21	Clay loam	34.7	No
**HH-36**	26.58	23.98	62.24	0.1	0.049	7.06	16	53	31	Silt loam	4.4x10^2	Yes
**HH-46**	16.28	43.63	226.84	ND[d]	ND[d]	6.82	ND[d]	ND[d]	ND[d]	ND[d]	1.3x10^3	Yes
**HH-50**	22.62	20.85	159.8	ND[d]	ND[d]	6.55	ND[d]	ND[d]	ND[d]	ND[d]	7.2x10^3	Yes

[a]Permanganate oxidizable active organic carbon.

[b]Particle size (mm) distribution according to USDA (%).

[c]Not applied.

[d]Not determined.

[e]Half the lower limit of detection (LOD).

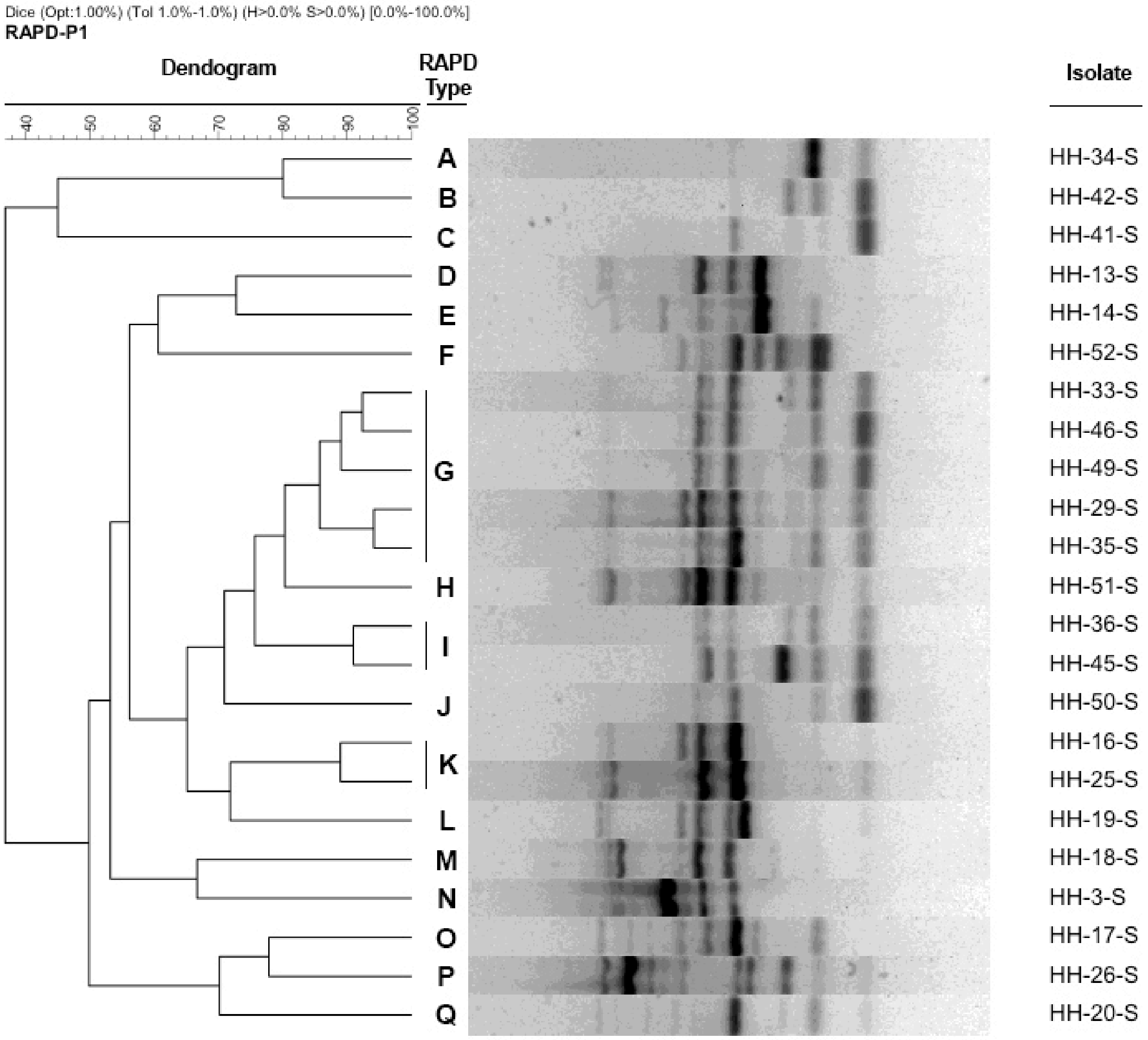

Figure S1. RAPD fingerprinting of 23 *E. coli* isolated from household soils of Mirzapur, Bangladesh. Dendogram (left) of the RAPD fingerprint (right) was constructed using the Bionumerics 4.5 software by Dice coefficient and the unweighted pair group method with arithmetic means (UPGMA). Distinct RAPD types (Dice coefficient >80%) are indicated with the letters A to Q.

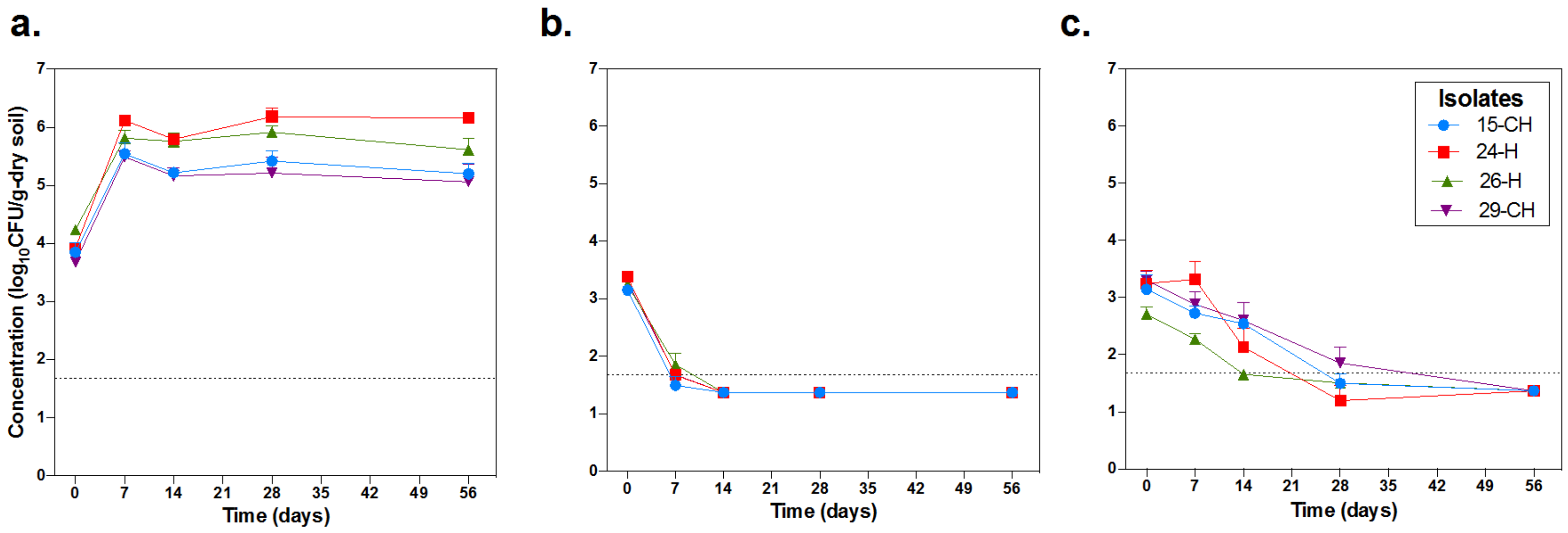

Figure S2. Survival dynamics of four *E. coli* isolates (15-CH, 24-H, 26-H, and 29-CH) in **(a)** autoclaved, **(b)** non-autoclaved and **(c)** a 1:1 ratio of autoclaved: non-autoclaved standard soil. In panel (c) the autoclaved fraction of the soil was seeded with *E. coli* and incubated for seven days before mixing with the non-autoclaved soil fraction. Each symbol represents the geometric mean $\log_{10}$ CFU per gram of dry soil and the error bar indicates the standard deviation of three independent replicates per isolate recovered at day 0, 7, 14, 28, and 56 after spiking the soil. Lower limit of detection (LOD) is indicated by the horizontal dotted line. When the CFU counts were below the lower LOD the value used to graph correspond to half the lower LOD.

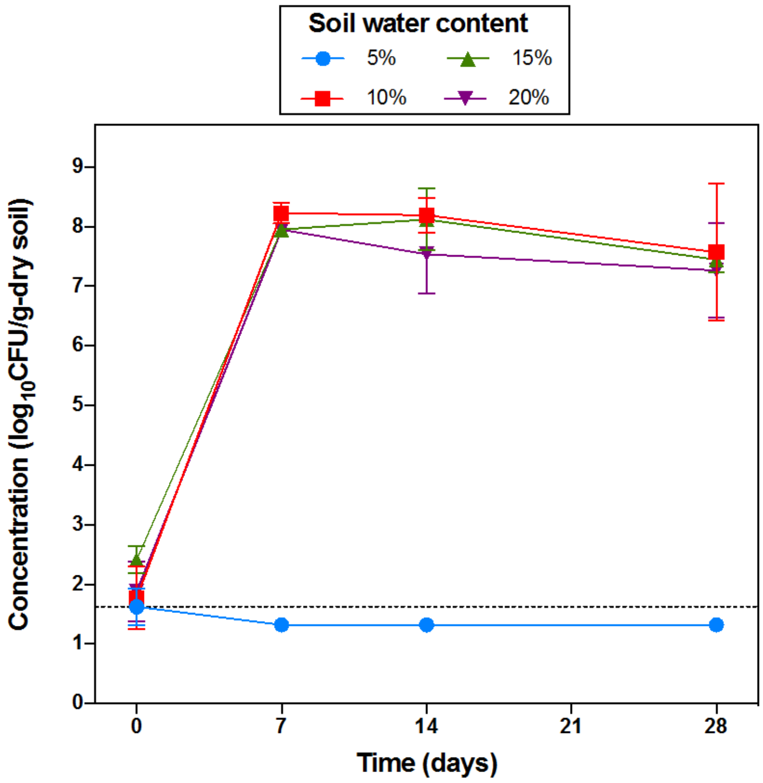

Figure S3. Survival dynamics of *E. coli* 26-H (isolated from human feces, classified as typical EPEC, resistant to third-generation cephalosporins, ESBL producer, and carrier of the CTX-M beta-lactamase) in autoclaved standard soil under different moisture conditions. Each symbol represents the geometric mean $\log_{10}$ CFU per gram of dry soil and the error bar indicates the standard deviation of three independent replicates per soil at day 0, 7, 14, and 28. The dotted line indicates the lower limit of detection (LOD). When the CFU counts were below the lower LOD the value used to graph correspond to half the lower LOD.
